# A Potent Anti-HB-EGF Monoclonal Antibody Inhibits Cancer Cell Proliferation and Multiple Angiogenic Activities of HB-EGF

**DOI:** 10.1371/journal.pone.0051964

**Published:** 2012-12-14

**Authors:** Shuji Sato, Andrew W. Drake, Isamu Tsuji, Jinhong Fan

**Affiliations:** 1 Takeda San Francisco, Inc., South San Francisco, California, United States of America; 2 Pharmaceutical Research Division, Takeda Pharmaceutical Company Limited, Fujisawa, Japan; Florida International University, United States of America

## Abstract

Heparin-binding epidermal growth factor-like growth factor (HB-EGF) is a member of the epidermal growth factor family and has a variety of physiological and pathological functions. Modulation of HB-EGF activity might have a therapeutic potential in the oncology area. We explored the therapeutic possibilities by characterizing the *in vitro* biological activity of anti-HB-EGF monoclonal antibody Y-142. EGF receptor (EGFR) ligand and species specificities of Y-142 were tested. Neutralizing activities of Y-142 against HB-EGF were evaluated in EGFR and ERBB4 signaling. Biological activities of Y-142 were assessed in cancer cell proliferation and angiogenesis assays and compared with the anti-EGFR antibody cetuximab, the HB-EGF inhibitor CRM197, and the anti-vascular endothelial growth factor (VEGF) antibody bevacizumab. The binding epitope was determined with alanine scanning. Y-142 recognized HB-EGF as well as the EGFR ligand amphiregulin, and bound specifically to human HB-EGF, but not to rodent HB-EGF. In addition, Y-142 neutralized HB-EGF-induced phosphorylation of EGFR and ERBB4, and blocked their downstream ERK1/2 and AKT signaling. We also found that Y-142 inhibited HB-EGF-induced cancer cell proliferation, endothelial cell proliferation, tube formation, and VEGF production more effectively than cetuximab and CRM197 and that Y-142 was superior to bevacizumab in the inhibition of HB-EGF-induced tube formation. Six amino acids in the EGF-like domain were identified as the Y-142 binding epitope. Among the six amino acids, the combination of F115 and Y123 determined the amphiregulin cross-reactivity and that F115 accounted for the species selectivity. Furthermore, it was suggested that the potent neutralizing activity of Y-142 was derived from its recognition of R142 and Y123 and its high affinity to HB-EGF. Y-142 has a potent HB-EGF neutralizing activity that modulates multiple biological activities of HB-EGF including cancer cell proliferation and angiogenic activities. Y-142 may have a potential to be developed into a therapeutic agent for the treatment of HB-EGF-dependent cancers.

## Introduction

Heparin-binding epidermal growth factor (EGF)-like growth factor (HB-EGF) is a member of the EGF family of growth factors that binds to the EGF receptor (EGFR) and ERBB4 [1], [2]. HB-EGF is synthesized as a membrane-bound form, proHB-EGF, which is known to be a juxtacrine growth factor [3], [4]. proHB-EGF undergoes ectodomain shedding by proteases [5], and the shedding is accelerated when proHB-EGF-expressing cells are exposed to certain stress conditions [6], [7]. The resulting soluble form of HB-EGF (sHB-EGF) has a potent mitogenic activity through the activation of EGFR [1]. Upon cleavage, the HB-EGF C-terminal fragment translocates into the nucleus and induces gene expression of cyclinA and cyclinD2 by suppressing the function of PLZF and Bcl6, respectively [8], [9].

Recent studies have revealed a variety of physiological functions of HB-EGF, including tissue development [10]–[12], skin wound healing [13], and pregnancy [14], [15]. HB-EGF has also been found to be associated with pathological processes, including cardiac hypertrophy [16], pulmonary hypertension [17], atherosclerosis [18], [19], and oncogenic transformation [20]. More recently, increasing evidence has demonstrated that HB-EGF is over-expressed in multiple types of cancers [21]–[25] and the over-expression has been shown to correlate with poor prognosis [24], [26], [27]. Due to these findings, anti-HB-EGF agents have been actively pursued for therapeutic applications. An HB-EGF inhibitor of the diphtheria toxin mutant, CRM197, is in Phase I clinical development for the treatment of advanced ovarian cancers [28]. Anti-HB-EGF antibodies U3-1565 and KHK2866 are currently in Phase I clinical trials for solid cancers [29]. An anti-HB-EGF therapeutic monoclonal antibody is expected to have a longer half-life compared to CRM197 [30], [31], but the generation of potent anti-HB-EGF antibodies has been challenging and few anti-HB-EGF monoclonal antibodies with a functional activity have been reported [29], [32], [33]. Recently, we reported the generation of neutralizing anti-HB-EGF monoclonal antibodies [34]. In this study, we report the characterization of one of the anti-HB-EGF monoclonal antibodies, Y-142, by analyzing its functional activities and binding epitope. The potent biological activity of Y-142 was compared with those of the anti-EGFR antibody cetuximab, of the HB-EGF inhibitor CRM197, and of anti-VEGF antibody bevacizumab.

## Materials and Methods

### Materials

Human, mouse, and rat sHB-EGF, and EGFR-hFc were previously prepared from the culture supernatant of 293F cells (Invitrogen) transfected with each expression plasmid [34]. EGFR ligands, anti-amphiregulin (anti-ARG) monoclonal antibody, anti-EGFR, anti-ERBB4, anti-HB-EGF, and anti-ARG polyclonal antibodies, FITC-labeled anti-CD31, anti-VEGF, biotinylated anti-VEGF, horseradish peroxidase-labeled (HRP-labeled) anti-phosphotyrosine antibodies were purchased from R&D Systems. Anti-phosphorylated ERK1/2 and anti-phosphorylated AKT antibodies were purchased from Cell Signaling Technology. Alexa488-labeled anti-rabbit IgG antibody was obtained from Invitrogen. Mouse control IgG, HRP-labeled streptavidin, HRP-labeled anti-mouse, anti-goat IgG antibodies, Cy5-labeled goat anti-mouse IgG Fcγ specific antibody, and anti-human IgG Fc antibody were purchased from Jackson ImmunoResearch Laboratories. Cetuximab and CRM197 were from ImClone and Sigma Aldrich, respectively. Sulfo-tagged anti-mouse antibody and sulfo-tagged streptavidin were purchased from Meso Scale Discovery. Sulfo-tagged anti-phosphotyrosine antibody was prepared by labeling anti-phosphotyrosine antibody (Millipore) with MSD Sulfo Tag reagent (Meso Scale Discovery).

### Antibody Generation

Anti-HB-EGF monoclonal antibody Y-142 was generated previously [34]. In brief, Y-142 was prepared by immunizing BALB/c mice (Japan Clea) with subcutaneous injections of keyhole limpet hemocyanin-conjugated sHB-EGF and abdominal injections of 293F cells transiently transfected with a proHB-EGF expression plasmid. Y-142 was purified from its hybridoma culture supernatant with rProteinA Sepharose (GE Healthcare). The animal study was carried out in strict accordance with the recommendations in the Guide for the Care and Use of Laboratory Animals of the National Institutes of Health. The protocol was approved by the Committee on the Ethics of Animal Experiments of Takeda Pharmaceutical Company Limited (Permit Number: 2802).

### Cell Culture

Ovarian cancer cell line SK-OV-3, breast cancer cell line T47D, and colorectal cancer cell line SW480 were purchased from American Type Culture Collection and maintained with McCoy’s 5A, RPMI1640, and Leibovit’z L-15 media supplemented with 10% serum, respectively. Normal human dermal fibroblasts (NHDF) and human umbilical vein endothelial cells (HUVEC) were purchased from Lonza and maintained with FGM-2 and EGM-2 kits (Lonza), respectively.

### Binding Specificity Test

Human, mouse, or rat sHB-EGF was immobilized on an MSD 384-well plate (Meso Scale Discovery). Non-specific binding was blocked with PBS containing 1% BSA. Y-142 was then added to each well and incubated for 1 hour at room temperature. Sulfo-tagged anti-mouse IgG antibody was added and incubated for 1 hour at room temperature. MSD Read Buffer T (Meso Scale Discovery) was added and chemiluminescence was measured with a Sector Imager 6000 (Meso Scale Discovery). EGFR ligand specificity of Y-142 was determined by incubating various concentrations of Y-142 for 1 hour in an EGFR ligand-immobilized 384-well plate followed by incubating HRP-labeled anti-mouse IgG antibody for 1 hour at room temperature. TMB Peroxidase EIA Substrate (BIORAD) was then added to the 384-well plate and the reaction was stopped after 15 minutes by adding 1N H_2_SO_4_. Antibody binding to EGFR ligand was then detected by measuring the absorbance at 450 nm using a SPECTRA MAX instrument (Molecular Devices).

### Biophysical Measurement of K_D_

KinExA experiments were performed using a KinExA 3200 instrument (Sapidyne) at 22°C. sHB-EGF was reconstituted into PBS. sHB-EGF and Y-142 samples were prepared in vacuum-degassed HBS-P buffer (10 mM HEPES, 150 mM NaCl, and 0.005% Tween-20) from GE Healthcare with filtered 0.01% BSA and 0.02% sodium azide. For the detection antibody, Cy5-labeled goat anti-mouse IgG, Fcγ specific was used. For each KinExA experiment, 20 µg of sHB-EGF was diluted into 1 mL of 50 mM sodium carbonate (pH 9.2) which was added directly to 50 mg of azlactone beads (UltraLink Biosupport, Thermo Scientific), and rocked overnight at 4°C. After rocking, the beads were rinsed once with 1 M Tris-HCl (pH 8.5) containing 1% BSA and rocked for 1 hour at room temperature in the same buffer. Coupled beads were added to the bead reservoir in the KinExA instrument and diluted to 30 mL with HBS-N (10 mM HEPES and 150 mM NaCl, GE Healthcare) containing 0.02% sodium azide which was also the running buffer for the KinExA instrument. All antigen-coupled beads were used immediately after preparation.

For K_D_-controlled experiments, 12 concentrations of sHB-EGF at a range of 4.04 fM–207 pM were equilibrated at room temperature for 72 hours with 1.03 pM Y-142 binding sites. The volume flowed through the bead pack for each sample in the K_D_-controlled titration was 23 mL at a flow rate of 0.25 mL/min. For antibody-controlled experiments, 12 concentrations of sHB-EGF at a range of 4.67 fM–239 pM were equilibrated at room temperature for 24 hours with 35.6 pM Y-142 binding sites. The volume flowed through the bead pack for each sample in the antibody-controlled titration was 3 mL at a flow rate of 0.25 mL/min. K_D_-controlled data and antibody-controlled data were simultaneously fit with a dual-curve positive cooperativity model using KinExA software (Version 3.13, Sapidyne).

### sHB-EGF Binding Inhibition to EGFR

The inhibitory activity of Y-142 against the binding of sHB-EGF to EGFR-hFc was detected as previously described [34]. In brief, anti-human IgG Fc antibody was immobilized onto a 96-well plate overnight at 4°C. After the plates were blocked with PBS containing 20% Immunoblock (Dainippon Sumitomo Pharma), EGFR-hFc was added and reacted for 1 hour at room temperature. Y-142 was then added and incubated at a concentration of 6.7 nM in the presence of 0.63 nM biotinylated sHB-EGF and 25 ng/mL of sodium heparin for 1 hour at 37°C, followed by adding HRP-labeled streptavidin and incubating for 1 hour at 37°C. SureBlue TMB Microwell Substrate was then added and the reaction was stopped after 15 minutes by adding 1N H_2_SO_4_. Absorbance at 450 nm was measured using SPECTRA MAX. The binding of sHB-EGF to EGFR-hFc in the presence of Y-142 was calculated as the percentage of the maximum binding that was measured of sHB-EGF binding to EGFR-hFc in the absence of Y-142. The minimum binding control signal was detected in the absence of sHB-EGF and Y-142.

### EGFR Phosphorylation Assay

EGFR phosphorylation was detected as described previously [34]. Briefly, SK-OV-3 cells were plated at 1×10^4^ cells/well in McCoy’s 5A medium containing 1% serum into a 96-well plate. After a 1-day culture, cells were incubated with 10 nM sHB-EGF or 10 nM ARG together with Y-142 or anti-ARG monoclonal antibody for 30 minutes at 37°C. Cells were lysed in Cell lysis buffer (Cell Signaling Technology) with a protease inhibitor cocktail (Roche Applied Science) and a phosphatase inhibitor cocktail (Sigma Aldrich). Cell lysates were then incubated in an anti-EGFR polyclonal antibody-coated plate for 1 hour at room temperature, followed by an incubation with HRP-labeled anti-phosphotyrosine antibody for 1 hour at room temperature. After an incubation with TMB Peroxidase EIA Substrate for 15 minutes, the reaction was stopped by adding 1N H_2_SO_4_. EGFR phosphorylation was detected by measuring the absorbance at 450 nm using an Envision plate reader (Perkin Elmer). EGFR phosphorylation in the presence of Y-142 or anti-ARG antibody was calculated as the percentage of the maximum EGFR phosphorylation measured in the absence of Y-142. The minimum phosphorylation control was signal detected in the absence of sHB-EGF, ARG, Y-142, and anti-ARG antibody.

### ERBB4 Phosphorylation Assay

T47D cells were seeded at 2.5×10^4^ cells/well into a 96-well plate with RPMI1640 medium containing 10% serum and cultured for 1 day. After being plated, the cells were serum-starved for 1 day and then treated with 10 nM sHB-EGF together with various concentrations of Y-142 for 30 minutes at 37°C. Cell lysates were prepared as in the EGFR phosphorylation assay described above and then incubated in an anti-ERBB4 polyclonal antibody-coated MSD 384-well plate for 1 hour at room temperature. To detect receptor phosphorylation, sulfo-tagged anti-phosphotyrosine antibody was incubated for 1 hour at room temperature. MSD Read Buffer T was then added as a substrate and chemiluminescence was measured with a Sector Imager 6000. ERBB4 phosphorylation in the presence of Y-142 was calculated as the percentage of the maximum ERBB4 phosphorylation measured in the absence of Y-142. The minimum phosphorylation control was signal detected in the absence of sHB-EGF and Y-142.

### ERK1/2 and AKT Phosphorylation Assays

For the detection of the phosphorylation of ERK1/2 or AKT, SK-OV-3 cells were plated as described above. The plated cells were then fixed with 3.8% paraformaldehyde for 1 hour at room temperature, washed with PBS containing 0.05% Tween-20 (wash buffer) three times, and blocked with wash buffer containing 1% BSA, 2% goat serum, 0.3 % cold fish skin gelatin, 0.1% TritonX-100, and 0.05% sodium azide. Cells were then incubated with anti-phosphorylated ERK1/2 antibody or anti-phosphorylated AKT antibody overnight at 4°C, washed three-times with wash buffer, and incubated with Alexa488-labeled anti-rabbit IgG antibody for 2 hours at room temperature. In order to measure total protein in each well, cells were incubated with Alexa647 succinimidyl ester (Invitrogen) in wash buffer. Phosphorylated ERK1/2 and AKT as well as total protein were detected with an ImageXpress Micro instrument (Molecular Devices). The levels of phosphorylation for ERK1/2 and AKT were normalized with the total amount of protein in each well. Phosphorylation levels were calculated as the percentage of the maximum phosphorylation levels detected in the absence of Y-142. The minimum control signal was detected in the absence of sHB-EGF and Y-142.

### Cell Proliferation Assay

SK-OV-3 cells were added at 3×10^3^ cells/well in McCoy’s 5A medium containing 1% serum and HUVEC were added at the same density in EBM-2 media (Lonza) containing 5% charcoal-stripped serum (Hyclone) to 96-well plates and cultured for 1 day. Cells were further cultured in the presence of 10 nM sHB-EGF with Y-142, cetuximab, or CRM197 for 3 days. Various concentrations of cetuximab and CRM197 were used in accordance with previous studies [35], [36]. Cell proliferation was detected with CellTiter-Glo (Promega) using Envision. Cell proliferation of SK-OV-3 cells and HUVEC was calculated as the percentage of the proliferation level measured in the absence of sHB-EGF, Y-142, cetuximab, and CRM197.

### Tube Formation Assay

NHDF were seeded at 1×10^4^ cells/well with an FGM-2 kit into a clear-bottom black 96-well plate and cultured for 3 days. One thousand HUVEC were seeded onto the monolayer of NHDF with EBM-2 medium containing 2% charcoal-stripped serum in the presence of 50 nM sHB-EGF with various concentrations of Y-142, cetuximab, CRM197, or bevacizumab. The broad concentration range of cetuximab, CRM197, or bevacizumab was used in accordance with previous studies [35]–[37]. After a 4-day incubation period, HUVEC were stained with FITC-labeled anti-CD31 antibody. CD31-positive cells were detected using an Acumen ex3 instrument (TTP Labtech). Tube formation in the presence of Y-142, cetuximab, CRM197, or bevacizumab was calculated as the percentage of the tube formation detected in the presence of sHB-EGF, Y-142, cetuximab, CRM197, and bevacizumab.

**Figure 1:**
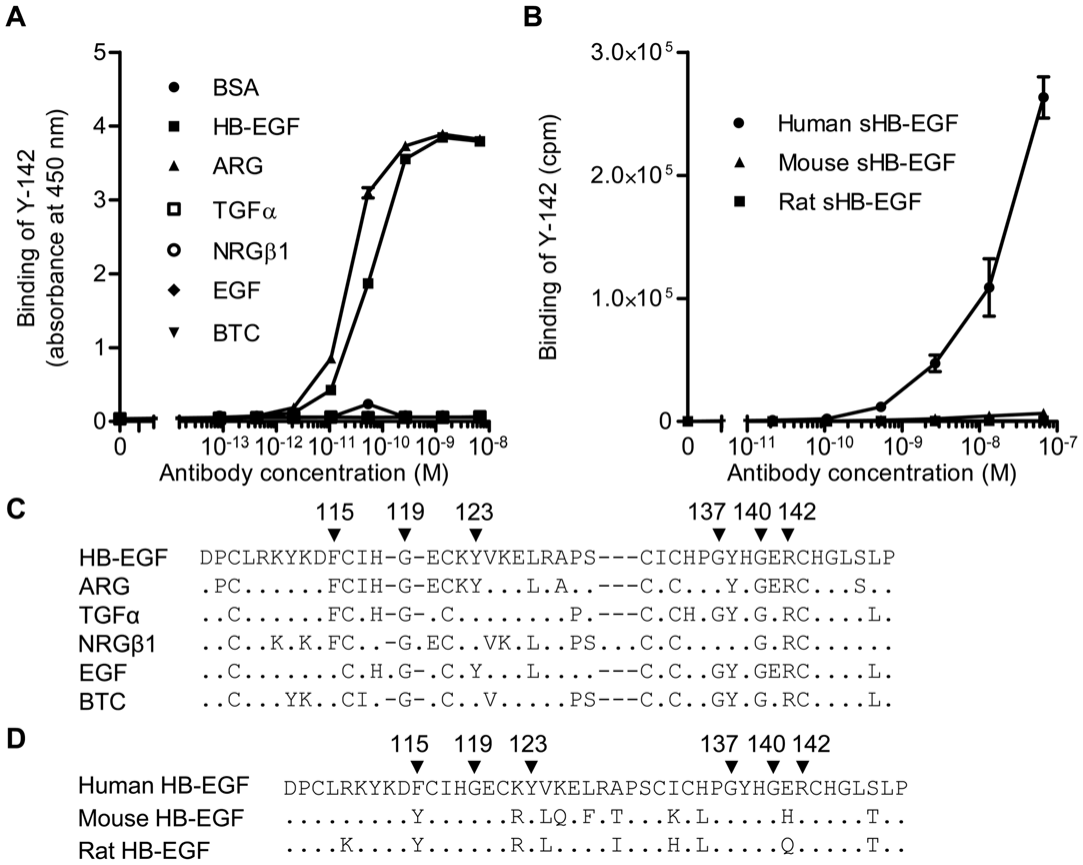
Binding specificity of Y-142 to EGFR ligands and to different species of sHB-EGF. (A) The binding activity of Y-142 to EGFR ligands by ELISA. The various concentrations of Y-142 were incubated in an EGFR ligand-immobilized plate. The binding was then detected with HRP-labeled anti-mouse IgG antibody. Data points represent the mean ± standard deviation (SD) of values acquired in duplicate. (B) The binding activity of Y-142 to human, mouse, and rat HB-EGF by ELISA. The binding activity to different species HB-EGF was measured by ELISA using an electroluminescence-based technology. The various concentrations of Y-142 were incubated in a sHB-EGF-immobilized plate. The binding was detected with sulfo-tagged anti-mouse IgG antibody. Data points represent the mean ± SD of values acquired in duplicate. (C) Amino acid alignment of the EGF-like domain of EGFR ligands. A dot indicates an amino acid different than HB-EGF. A dash represents a gap. Arrowheads labeled with a number indicate the Y-142 binding epitopes identified in Fig. 6. (D) Amino acid alignment of the EGF-like domain of human, mouse, and rat HB-EGF. A dot indicates an amino acid identical to human HB-EGF. Arrowheads labeled with a number indicate the Y-142 binding epitopes identified in Fig. 6.

### VEGF Measurement

Anti-VEGF monoclonal antibody was immobilized in an MSD high binding plate overnight at 4°C. Each well was then blocked with PBS containing 1% BSA for 1 hour at room temperature. Culture supernatant from the tube formation assay described above was then added into each well and the plate was incubated for 1 hour at room temperature. Biotinylated anti-VEGF antibody was then reacted for 1 hour at room temperature followed by an incubation with sulfo-tagged streptavidin for 1 hour at room temperature. Read T Buffer was then added as a substrate and chemiluminescence was measured with a Sector Imager 6000 instrument. The VEGF amount in the culture supernatant was calculated as a percentage of the VEGF amount in the presence of sHB-EGF.

### Western Blot

sHB-EGF and ARG were boiled in laemmli sample buffer (BIORAD) with or without 10 mM dithiothreitol for 5 minutes at 95°C. The non-reduced or reduced sHB-EGF or ARG were then subjected to sodium dodecyl sulfate-polyacrylamide gel electrophoresis. sHB-EGF was detected with either 3 µg/mL of Y-142 or 3 µg/mL of anti-HB-EGF polyclonal antibody as the primary antibody, followed by incubation with HRP-labeled anti-mouse IgG antibody or HRP-labeled anti-goat IgG antibody, respectively, as the secondary antibody. ARG was detected with either 3 µg/mL of Y-142 or 3 µg/mL of anti-ARG polyclonal antibody as the primary antibody, followed by incubation with HRP-labeled anti-mouse IgG antibody or HRP-labeled anti-goat IgG antibody, respectively, as the secondary antibody.

### Epitope Mapping

Epitope mapping study was performed as described previously [33]. In brief, expression plasmids of proHB-EGF alanine mutants were prepared with a KOD Plus Mutagenesis Kit (TOYOBO). Each expression plasmid was transfected into SW480 cells with Lipofectamine LTX with Plus reagent (Invitrogen) in 96-well plates. Two days after the transfection, cells were washed once with PBS(+) (PBS with 0.5 mM CaCl_2_ and 0.5 mM MgCl_2_) and then incubated in 1% BSA-containing PBS for 30 minutes at 4°C. Cells were then washed three times with PBS(+) and incubated with 200 nM Y-142 or anti-HB-EGF polyclonal antibody for 30 minutes at 4°C. After the washing steps, HRP-labeled anti-mouse IgG or HRP-labeled anti-goat IgG-antibody was added to detect Y-142 or anti-HB-EGF polyclonal antibody, respectively. After washing twice with PBS(+), TMB Peroxidase EIA Substrate was added to each well and incubated for 15 minutes. The reaction was stopped by adding 1N H_2_SO_4_. Antibody binding was detected by measuring the absorbance at 450 nm using an Envision instrument. In order to take into consideration the differences among proHB-EGF expression levels, the binding of Y-142 to each proHB-EGF mutant was normalized with that of the anti-HB-EGF polyclonal antibody by each mutant. The percent binding of Y-142 was then calculated using the following formula: Y-142 binding (%)  =  (A/B)/(C/D)×100, where A represents the absorbance at 450 nm of Y-142 in mutant proHB-EGF, B represents the absorbance (450 nm) of anti-HB-EGF polyclonal antibody in mutant proHB-EGF, C represents the absorbance (450 nm) of Y-142 in wild-type proHB-EGF, and D represents the absorbance (450 nm) of anti-HB-EGF polyclonal antibody in wild-type proHB-EGF.

## Results

### Binding Specificity of Y-142

Neutralizing anti-HB-EGF antibodies were previously generated by a hybridoma approach [34]. In the study, we characterized one of the anti-HB-EGF monoclonal antibodies, Y-142. We first tested the binding profile of Y-142 to EGF ligands using ELISA (Fig. 1A). Y-142 showed comparable binding to HB-EGF and amphiregulin (ARG), but not to the other four EGFR ligands. We then examined species specificity of Y-142 by testing its binding to human, mouse and rat HB-EGF. As shown in Fig. 1B, Y-142 bound to human sHB-EGF but not to mouse and rat HB-EGF.

### Biophysical Measurement of K_D_ for Y-142 to HB-EGF

**Figure 2:**
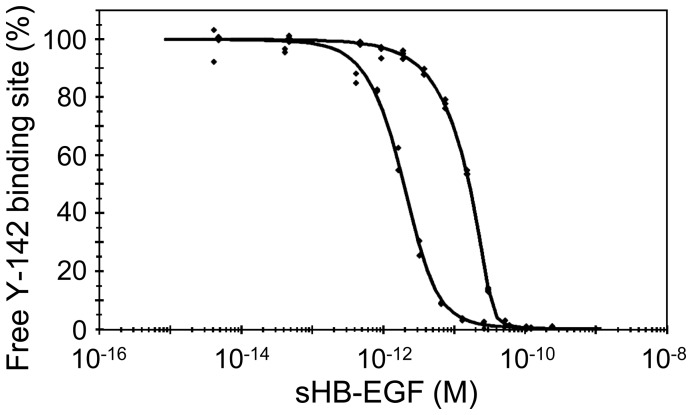
Measuring the K_D_ of the Y-142/HB-EGF complex. Dual-curve KinExA equilibrium titration of sHB-EGF binding to Y-142. K_D_-controlled data (bottom fitted curve) were acquired by equilibrating sHB-EGF at a concentration range of 4.04 fM–207 pM with 1.03 pM Y-142 binding sites. Antibody-controlled data (top fitted curve) were acquired by equilibrating sHB-EGF at a concentration range of 4.67 fM–239 pM with 35.6 pM Y-142 binding sites. All data points were acquired in duplicate. Both curves were simultaneously fit to a standard positive cooperativity equilibrium model, yielding an effective K_D_ = 1.50 pM (0.31) where the number in parentheses is the 95% confidence interval of the fit, and a Hill coefficient n = 1.68.

The K_D_ value of Y-142 to human HB-EGF was measured using the kinetic exclusion assay (KinExA) method. K_D_-controlled titration data were obtained by using a range of sHB-EGF concentrations equilibrated with a constant Y-142 binding site concentration (2× the molecular concentration) of 1.03 pM. For antibody-controlled experiments, several concentrations of sHB-EGF were equilibrated with a constant Y-142 binding site concentration of 35.6 pM. In a dual-curve analysis, the K_D_-controlled curve contains most of the K_D_ information while the antibody-controlled curve returns a value for the binding site concentration of the monoclonal antibody. The latter parameter can be compared to the nominal binding site concentration of the monoclonal antibody which can determine if the estimated K_D_ should be adjusted for the activity of the antigen in certain cases [38]. The K_D_-controlled titration data and antibody-controlled titration data were initially fit in a dual-curve analysis with a standard 1∶1 equilibrium binding model. It was observed, however, that the titration curves collected under both K_D_- and antibody-controlled conditions decreased with a slope steeper than that described by the standard 1∶1 model. This steeper slope can only be explained by use of a positive cooperativity model. In the positive cooperativity model, the binding of HB-EGF to one monoclonal antibody binding site causes the affinity of the second binding site of the antibody to increase [39]. Hence, a positive cooperativity equilibrium model was used to fit the dual-curve titration data which provided an improved fit to the dual curve data set, yielding an effective K_D_  =  1.50 pM (Fig. 2). In addition, the resulting Hill coefficient (n  =  1.68) was greater than 1 which also indicated positive cooperativity (n  =  1 signifies independent binding) [39].

### Neutralizing Activity of Y-142 against sHB-EGF and ARG Signaling

**Figure 3:**
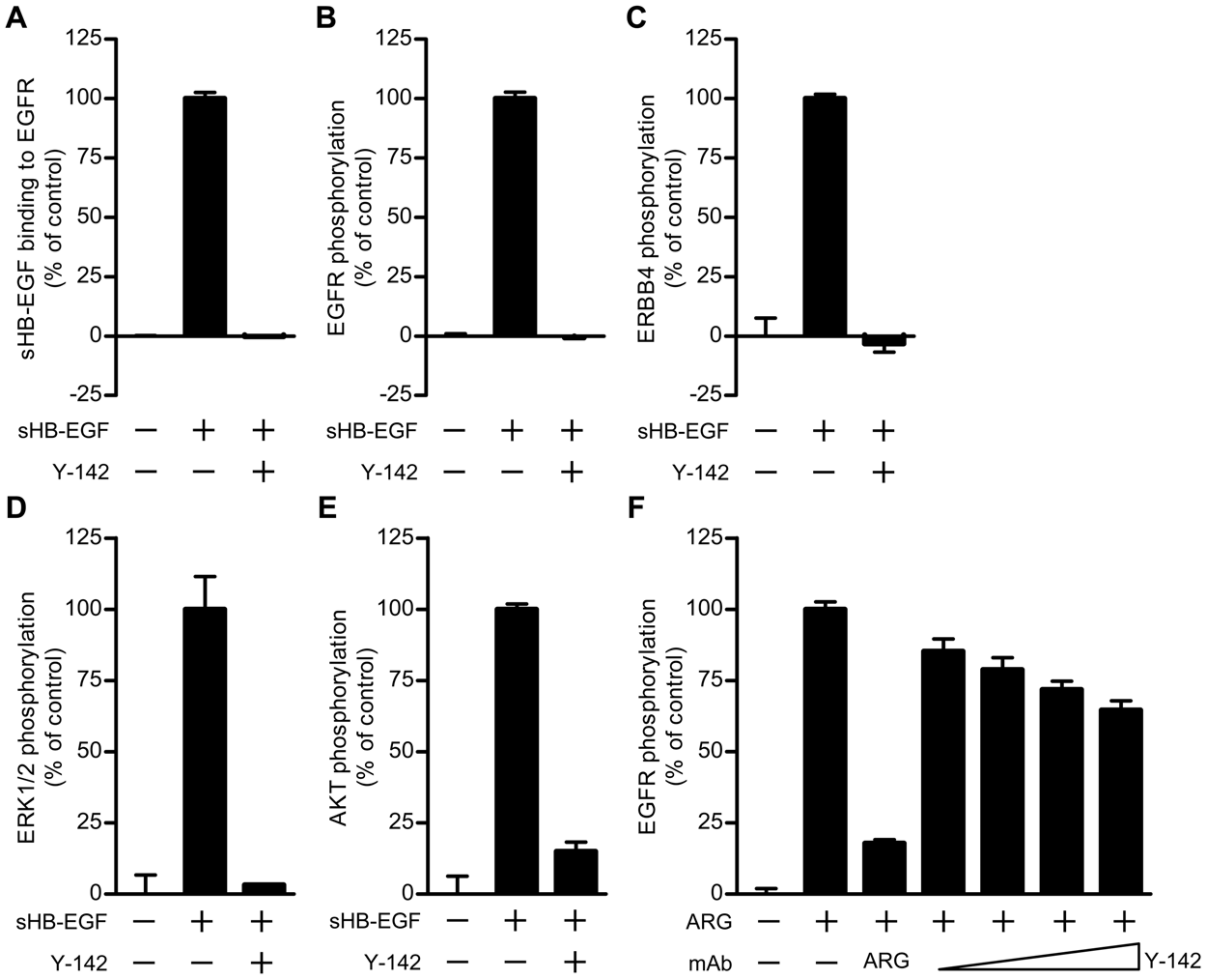
Neutralizing activities of Y-142 against sHB-EGF and ARG signaling. (A) Inhibitory activity of Y-142 to sHB-EGF binding to EGFR. EGFR-hFc was incubated in an anti-human IgG Fc antibody-coated plate. Y-142 was then incubated at a concentration of 6.7 nM in the presence of 0.63 nM biotinylated sHB-EGF for 1 hour at 37°C. sHB-EGF bound to EGFR-hFc was detected by HRP-labeled streptavidin. sHB-EGF binding to EGFR-hFc in the presence of Y-142 was calculated as a percentage of the “control” sHB-EGF binding to EGFR which occurred without Y-142. Data points represent the mean + SD of values acquired in triplicate. (B) Neutralizing activity of Y-142 against EGFR phosphorylation. SK-OV-3 cells were treated with 10 nM sHB-EGF and 67 nM Y-142. Cell lysates were incubated in an anti-EGFR antibody-coated plate, followed by an incubation with HRP-labeled anti-phosphorytosine antibody. EGFR phosphorylation in the presence of Y-142 was calculated as a percentage of the “control” EGFR phosphorylation which occurred without Y-142. Data points represent the mean + SD of values acquired in triplicate. (C) Neutralizing activity of Y-142 against ERBB4 phosphorylation. Cell lysates of T47D cells as prepared in Fig. 3A were incubated on an anti-ERBB4 antibody-coated plate. The phosphorylation of ERBB4 was detected by a sulfo-tagged anti-phosphotyrosine antibody. ERBB4 phosphorylation in the presence of Y-142 was calculated as a percentage of the “control” ERBB4 phosphorylation which occurred without Y-142. Data points represent the mean + SD of values acquired in duplicate. (D) and (E) Neutralizing activity of Y-142 against (D) ERK1/2 phosphorylation and (E) AKT phosphorylation. In (D) and (E) SK-OV-3 cells treated with 10 nM sHB-EGF and 200 nM Y-142 were stained with an anti-phosphorylated ERK1/2 antibody or an anti-phosphorylated AKT antibody, respectively, followed by an Alexa488-labeled anti-rabbit IgG antibody. Phosphorylated ERK1/2 and phosphorylated AKT were both detected with an ImageXpress Micro instrument and calculated as a percentage of the “control” phosphorylation levels which occurred without Y-142. Data points represent the mean + SD of values acquired in duplicate. (F) Neutralizing activity of Y-142 to ARG. SK-OV-3 cells were treated with 10 nM ARG plus various concentrations of Y-142 (2 nM, 6.7 nM, 20 nM, and 67 nM). Anti-ARG monoclonal antibody (67 nM) was used as a positive control. Cell lysates were incubated in an anti-EGFR antibody-coated plate followed by an incubation with an HRP-labeled anti-phosphorytosine antibody. EGFR phosphorylation was calculated as a percentage of the “control” EGFR phosphorylation which occurred without Y-142. Data points represent the mean + SD of values acquired in triplicate.

**Figure 4:**
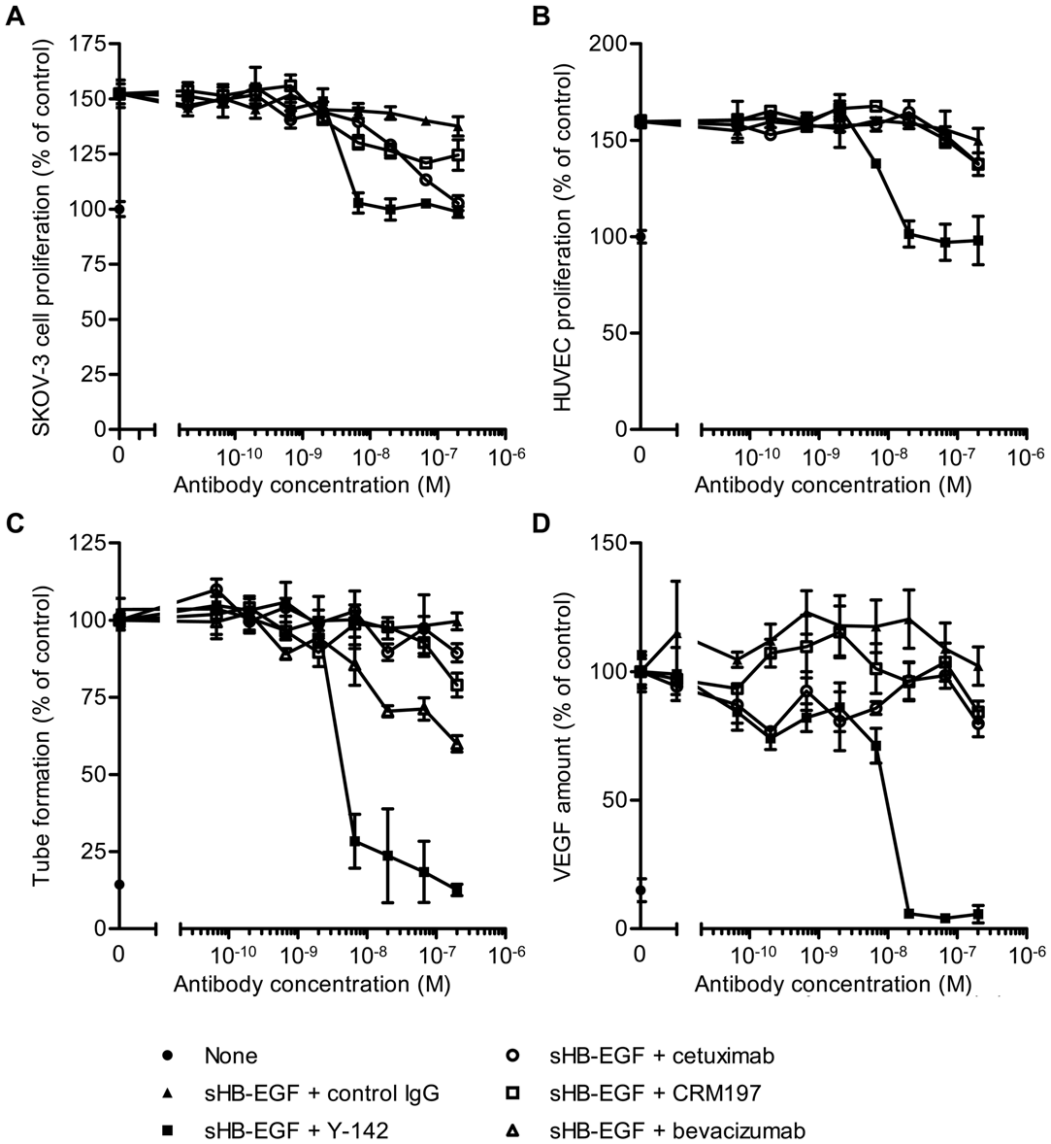
Inhibitory activity of Y-142 against sHB-EGF functions. (A) and (B) Neutralizing activities of Y-142 against (A) sHB-EGF-induced SK-OV-3 cell proliferation and (B) HUVEC proliferation. SK-OV-3 cells or HUVEC were cultured for 3 days in the presence of sHB-EGF and the indicated concentrations of Y142, cetuximab, or CRM197. Cell proliferation was detected with CellTiter-Glo and calculated as a percentage of the “control” cell proliferation without sHB-EGF. Data points represent the mean ± SD of values acquired in triplicate. (C) Inhibition of HUVEC tube formation by Y-142. HUVEC were cultured on a monolayer of NHDF in the presence of 50 nM sHB-EGF and the indicated concentrations of Y142, cetuximab, CRM197, or bevacizumab for 4 days. HUVEC were then stained with FITC-labeled anti-CD31 antibody. Tube formation (CD31-positive area) was calculated as a percentage of the “control” amount of tube formation in the presence of sHB-EGF. Data points represent the mean ± SD of values acquired in triplicate. (D) Inhibition of VEGF production by Y-142. HUVEC were prepared as in Figure 4C and treated with 50 nM sHB-EGF and the indicated concentrations of Y142, cetuximab, or CRM197 for 4 days. VEGF concentration in the supernatant of co-culture was measured in an electrochemiluminescence-based method. VEGF production was calculated as a percentage of the “control” amount of VEGF produced in the presence of sHB-EGF. Data points represent the mean ± SD of values acquired in triplicate.

**Figure 5:**
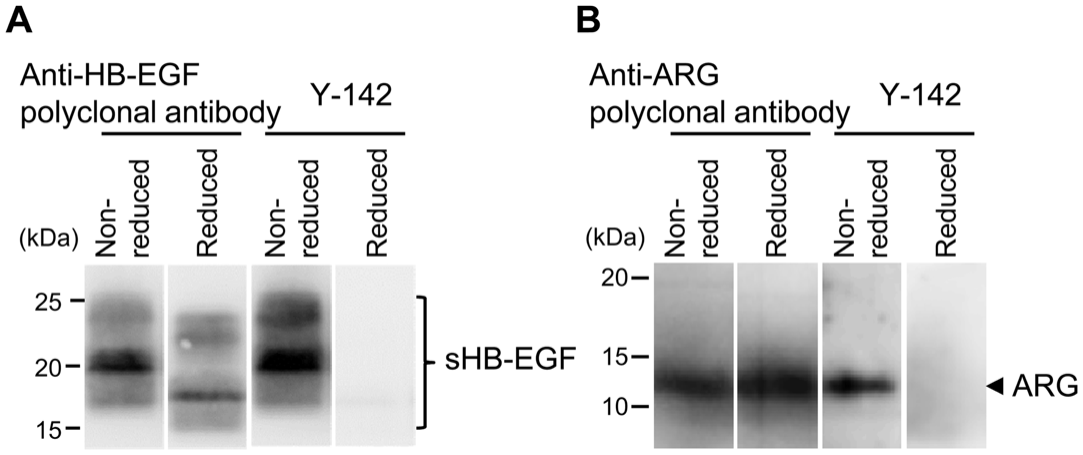
Recognition of a conformational epitope by Y-142. The binding of Y-142 to a linear or conformational epitope was tested using Western blot. sHB-EGF (A) or ARG (B) was prepared in reducing or non-reducing conditions with or without dithiothreitol, respectively. The sHB-EGF was probed with an anti-HB-EGF polyclonal antibody and with Y-142. The ARG was probed with anti-ARG polyclonal antibody and with Y-142.

**Figure 6:**
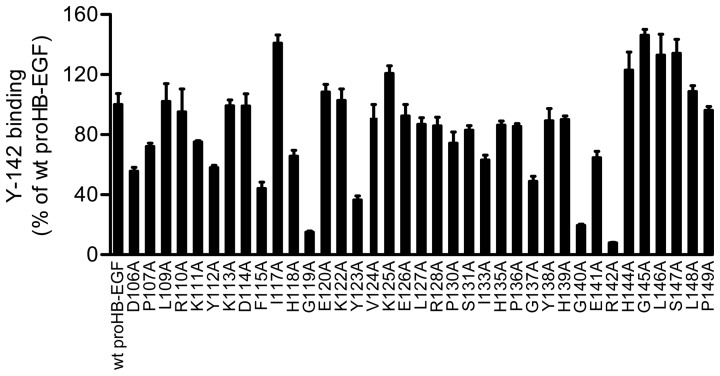
Recognition of F115, G119, Y123, G137, G140, and R142 by Y-142. Epitope mapping of Y-142 was performed using alanine scanning. Each mutant proHB-EGF expression plasmid was transfected into SW480 cells. The binding activity of Y-142 to the cells was measured in a cell ELISA. The expression level of mutant proHB-EGF was normalized with the binding of anti-HB-EGF polyclonal antibody by each mutant. The binding of Y-142 to mutant proHB-EGF was calculated as a percentage of the “control” binding of Y-142 to wild-type proHB-EGF. Data points represent the mean + SD of values acquired in triplicate.

As the over-expression of HB-EGF has been reported in cancer tissues [21]–[25], the neutralization of sHB-EGF functionality is expected as a promising therapeutic potential. Neutralizing activity of Y-142 against sHB-EGF was therefore evaluated in both biochemical and cell-based assays. EGFR is one of the HB-EGF receptors, and the binding of sHB-EGF to EGFR leads to phosphorylation of EGFR and activation of its downstream signaling. We found that the binding of sHB-EGF to EGFR-hFc was completely blocked with Y-142 (Fig. 3A). The sHB-EGF blocking activity of Y-142 was translated with the complete inhibition of sHB-EGF-induced EGFR phosphorylation (Fig. 3B). In addition to EGFR, sHB-EGF binds to and activates ERBB4 signaling pathway [2]; indeed we showed that Y-142 could neutralize the sHB-EGF-induced phosphorylation of endogenously expressed ERBB4 on T47D cells (Fig. 3C). Importantly, the neutralizing activity of Y-142 affected EGFR downstream signaling events. As shown in Figs. 3D and 3E, Y-142 neutralized sHB-EGF-induced phosphorylation of ERK1/2 and AKT, respectively. Taken together, our results showed that sHB-EGF binds to and activates EGFR and ERBB4, and that Y-142 can neutralize sHB-EGF-induced EGFR and ERBB4 signaling.

In a binding specificity test, Y-142 recognized ARG as well as sHB-EGF, which are both EGFR ligands (Fig. 1A). We then tested if Y-142 could neutralize the biological activity of ARG. We hypothesized that Y-142 would be able to neutralize the functionality of ARG because of its neutralizing activity against sHB-EGF and cross-reactivity to ARG. However, we were able to show that phosphorylation of EGFR induced by ARG was only partially neutralized by Y-142 (Fig. 3F), whereas Y-142 completely blocked the sHB-EGF-induced EGFR phosphorylation (Fig. 3B). These results suggested that Y-142 could neutralize both sHB-EGF and ARG functional activities, albeit ARG activity on EGFR is only partially blocked by Y-142.

### Comparison of sHB-EGF Neutralizing Activity of Y-142 with those of Cetuximab, CRM197, and Bevacizumab

The neutralizing activity of Y-142 against sHB-EGF was compared with two known inhibitors of the EGFR pathway: cetuximab and CRM197. Cetuximab, an anti-EGFR monoclonal antibody used as a cancer therapeutic agent, suppresses EGFR-dependent cancer cell growth by inhibiting EGFR activation. CRM197, a mutant diphtheria toxin, binds to proHB-EGF and the subsequent internalization of CRM197 causes the inhibition of protein synthesis. We observed that sHB-EGF-induced SK-OV-3 cell proliferation was completely inhibited by Y-142 and cetuximab, and partially inhibited by CRM197 (Fig. 4A). Y-142 showed a more potent effect in suppressing cell proliferation than cetuximab. IC_50_ values of Y-142 and cetuximab were 4.1 nM and 38 nM, respectively.

sHB-EGF has been reported to be involved in multiple processes of angiogenesis [40], [41]. We therefore studied the effect of Y-142 in a human umbilical vein endothelial cells (HUVEC) proliferation assay. Y-142 neutralized sHB-EGF-induced HUVEC proliferation in a concentration dependent manner. Conversely, in the same assay, cetuximab and CRM197 showed no significant activity (Fig. 4B). To confirm this initial observation, we investigated the ability of Y-142, cetuximab, and CRM197 to block the angiogenic activity of sHB-EGF in a tube formation assay in which HUVEC and normal human dermal fibroblasts (NHDF) were co-cultured. Y-142 showed a complete inhibition of sHB-EGF-induced tube formation while cetuximab and CRM197 had no significant effect (Fig. 4C). To further evaluate the role of Y-142 in the angiogenic activity of sHB-EGF, we tested the neutralizing activity of Y-142 against sHB-EGF-induced VEGF production. We observed that Y-142 inhibited VEGF production induced by sHB-EGF while cetuximab and CRM197 showed no inhibitory effects (Fig. 4D). These results indicate that Y-142 could neutralize the angiogenic processes of sHB-EGF more effectively than cetuximab and CRM197.

We further compared the inhibitory activity of Y-142 against the sHB-EGF-induced tube formation with that of anti-VEGF antibody bevacizumab, which is used as a cancer therapeutic agent. As shown in Fig. 4C, bevacizumab only partially inhibited the sHB-EGF-induced tube formation, while Y-142 showed complete inhibition.

### Conformational Epitope Recognition and Epitope Mapping of Y-142

Our results show that Y-142 binds to HB-EGF and ARG and blocks the binding of sHB-EGF to EGFR and ERBB4 and ARG to EGFR. These findings suggest that Y-142 recognizes the EGF-like domain of HB-EGF because the EGF-like domain is required for the interaction of the EGF family of ligands and receptors. When probing sHB-EGF by Western blot under reducing and non-reducing conditions, Y-142 only recognized sHB-EGF under non-reducing condition, suggesting that it might recognize a conformational epitope (Fig. 5A). Because Y-142 recognized ARG as well as sHB-EGF in the specificity test (Fig. 1A), we tested the recognition pattern of Y-142 to ARG. Similarly to sHB-EGF, Y-142 only recognized ARG under non-reducing condition (Fig. 5B), further supporting that Y-142 recognizes a conformational epitope. To identify the Y-142 epitope, we tested its binding activity against a series of proHB-EGF mutants where an alanine point mutation was introduced in the EGF-like domain. Six cysteines in this domain were not replaced so as not to disrupt the disulfide bonds required for EGFR activation [42]. Data demonstrated that the binding of Y-142 to G119A, G140A, and R142A mutants decreased by more than 70%, while binding to F115A, Y123A, and G137A mutants decreased by more than 50% (Fig. 6). These results indicate that Y-142 recognizes F115, G119, Y123, G137, G140, and R142 in the EGF-like domain of HB-EGF.

## Discussion

In this study, we characterized an anti-HB-EGF monoclonal antibody Y-142 and evaluated its therapeutic potential. Our results clearly demonstrated that Y-142 inhibits sHB-EGF-induced cancer cell proliferation as well as sHB-EGF-induced angiogenic processes more effectively than cetuximab and CRM197, suggesting that Y-142 may have more promising therapeutic possibilities than cetuximab and CRM197. We hypothesized that the unique epitope of Y-142 and its high affinity to HB-EGF accounted for its superior activities in blocking cell proliferation and angiogenic activities of sHB-EGF. Previous studies have reported that the K_D_ value of HB-EGF binding to EGFR is 3.8 nM [43], the K_D_ of CRM197 binding to HB-EGF is 27 nM [44], and that the K_D_ of cetuximab binding to EGFR is 0.2 nM [45]. Compared to these reported affinities, the K_D_ of Y-142 binding to HB-EGF measured in this study (1.5 pM) was several orders of magnitude tighter (Fig. 2). Because Y-142 and CRM197 bind to HB-EGF and cetuximab binds to EGFR, it is difficult to directly compare the amino acids used for binding by each reagent to inhibit the sHB-EGF-EGFR interaction. However, we expect that their binding amino acids should be another determinant of their neutralizing activities. More interestingly, the inhibitory activity of Y-142 seemed to be enhanced in the HUVEC proliferation and tube formation assays compared to that in the cancer cell proliferation assay (Figs. 4A, 4B and 4C). As shown in Fig. 4C, the sHB-EGF-induced tube formation was only partially blocked by bevacizumab, implying that sHB-EGF uses VEGF-independent and VEGF-dependent pathways. Therefore, we speculate that the complete inhibition of Y-142 against the function of sHB-EGF, which led to the subsequent inhibition of the VEGF function, resulted in the enhanced activity of Y-142.

To date, few anti-HB-EGF antibodies with biological activity against HB-EGF have been reported in the literature [29], [32], [33]. However, the results of this study show that Y-142 has unique properties with its potent neutralizing activity in multiple HB-EGF signaling events. We compared the neutralizing activity of Y-142 with that of KM3566, which is a parental antibody of KHK2866 [29]. KHK2866 is the humanized version of mouse anti-HB-EGF antibody KM3566. The IC_50_ value of KM3566 was estimated to be approximately 0.2 µg/mL (1.3 nM) in an MCAS cell growth assay using 3 ng/mL (0.32 nM) of sHB-EGF [29]. This estimated IC_50_ value was more than four-fold higher than the concentration of sHB-EGF used. In contrast, the IC_50_ value of Y-142 was 4.3 nM in an SK-OV-3 cell growth assay using 10 nM sHB-EGF (Fig. 4A). We previously identified the IC_50_ values of anti-HB-EGF monoclonal antibodies with a colony formation assay [34]. In the assay, Y-142 showed an IC_50_ value of 0.02 nM against 0.11 nM sHB-EGF. Hence, each assay showed the IC_50_ values of Y-142 were less than half the concentrations of sHB-EGF used, indicating a superior neutralization activity of Y-142 compared to KM3566.

Our study also shed light on the functional epitope of Y-142. An alanine scanning approach to the whole EGF-like domain revealed six amino acids, F115, G119, Y123, G137, G140, and R142, as the Y-142 binding epitope (Fig. 6). This mapping result is consistent with the finding that Y-142 did not recognize a linear conformational epitope (Fig. 5). Structural analyses by nuclear magnetic resonance and crystallography demonstrated that Y13 and R41 of EGF, corresponding to F115 and R142 of HB-EGF (Fig. 1C), were in close proximity to each other [46], [47]. Mutational analyses of EGF identified Y13, I23, R41, and L47 of EGF as crucial amino acids in its binding to EGFR [48]–[50]. R41 in EGF is an especially critical determinant for EGFR binding [48] because it forms a salt bridge with D355 in domain III of EGFR [46]. Y13 in EGF was reported to hydrophobically interact with the F357 side chain in domain III of EGFR [47]. Consistent with the mutagenesis data of EGF, the neutralizing activity of Y-142 against sHB-EGF was predicted to be attributed to the recognition of F115 and R142. In addition to the binding studies of EGF to EGFR, mutagenesis approaches with heregulinβ (HRGβ), a ligand for ERBB4, identified several residues critical for ERBB4 binding [51]. Replacement of R44 in HRGβ with an alanine showed the greatest reduction of ERBB4 binding. Furthermore, the replacement of F13, G18, and G42 in HRGβ also resulted in an apparent reduction in ERBB4 binding. These amino acids in HRGβ correspond to R142, F115, G119, and G140 in HB-EGF, respectively (Fig. 1C). Similar to the binding between HRGβ and ERBB4, the binding activity of Y-142 to HB-EGF was reduced when R142, F115, G119, and G140 in HB-EGF were mutated. The HRGβ mutagenesis approach was consistent with the model that describes the neutralizing activity of Y-142 to ERBB4 as being attributed to the recognition of these amino acids on HB-EGF.

Species specificity and EGFR ligand specificity provided additional information of the epitope of Y-142. Of the six amino acids determined to be the Y-142 binding epitope (F115, G119, Y123, G137, G140, R142), all except for F115 were conserved between human, mouse, and rat HB-EGF (Fig. 1D), yet Y-142 binds to human HB-EGF specifically. This indicates that human HB-EGF specificity was determined by F115. Among the six amino acids which define the Y-142 epitope, F115 and Y123 are the only two which are shared between ARG and HB-EGF and not with other EGF ligands (Fig. 1C). Therefore, we concluded that the cross-reactivity of Y-142 to ARG resulted from the recognition of the combination of F115 and Y123. Among EGF ligands, ARG and HB-EGF share common features. Both ARG and HB-EGF possess a heparin-binding domain [52] and bind to CD9, which potentiates their juxtacrine activities [53]. In our studies, however, Y-142 only partially neutralized ARG (Fig. 3F), whereas sHB-EGF-induced EGFR phosphorylation was completely neutralized by Y-142 (Fig. 3B). Of the six amino acids identified as the Y-142 epitope, G137 was the only amino acid not shared with ARG. These findings may mean that the recognition of G137 of Y-142 may also be important in its neutralizing activity against sHB-EGF. Interestingly, the co-expression of ARG and HB-EGF in gastrointestinal stromal tumor tissues and breast cancer tissues has been reported [54], [55]. In addition, the co-expression has been identified in cancer cell lines derived from different cancer types including bladder, head and neck squamous, prostate, and ovarian cancers [56]–[59], suggesting that Y-142 may be able to exert its synergistic anti-cancer activity against these cancer types by neutralizing sHB-EGF and ARG functionalities simultaneously.

We demonstrated the potent neutralizing activity of Y-142 against sHB-EGF and the superiority of Y-142 over cetuximab, CRM197, and bevacizumab in sHB-EGF neutralizing activities. Our findings may expedite progress in the clinical research of HB-EGF.

## References

[1] Higashiyama S, Abraham JA, Miller J, Fiddes JC, Klagsbrun M (1991) A heparin-binding growth factor secreted by macrophage-like cells that is related to EGF. Science 251: 936–939. 1840698 10.1126/science.1840698

[2] Elenius K, Paul S, Allison G, Sun J, Klagsbrun M (1997) Activation of HER4 by heparin-binding EGF-like growth factor stimulates chemotaxis but not proliferation. EMBO J 16: 1268–1278. 9135143 10.1093/emboj/16.6.1268 PMC1169725

[3] Higashiyama S, Lau K, Besner GE, Abraham JA, Klagsbrun M (1992) Structure of heparin-binding EGF-like growth factor. Multiple forms, primary structure, and glycosylation of the mature protein. J Biol Chem 267: 6205–6212. 1556128

[4] Higashiyama S, Iwamoto R, Goishi K, Raab G, Taniguchi N, et al (1995) The membrane protein CD9/DRAP 27 potentiates the juxtacrine growth factor activity of the membrane-anchored heparin-binding EGF-like growth factor. J Cell Biol 128: 929–938. 7876316 10.1083/jcb.128.5.929 PMC2120393

[5] Shiomi T, Lemaître V, D’Armiento J, Okada Y (2010) Matrix metalloproteinases, a disintegrin and metalloproteinases, and a disintegrin and metalloproteinases with thrombospondin motifs in non-neoplastic diseases. Pathol Int 60: 477–496. 20594269 10.1111/j.1440-1827.2010.02547.x PMC3745773

[6] Goishi K, Higashiyama S, Klagsbrun M, Nakano N, Umata T, et al (1995) Phorbol ester induces the rapid processing of cell surface heparin-binding EGF-like growth factor: conversion from juxtacrine to paracrine growth factor activity. Mol Biol Cell 6: 967–980. 7579712 10.1091/mbc.6.8.967 PMC301256

[7] Takenobu H, Yamazaki A, Hirata M, Umata T, Mekada E (2003) The stress- and inflammatory cytokine-induced ectodomain shedding of heparin-binding epidermal growth factor-like growth factor is mediated by p38 MAPK, distinct from the 12-O-tetradecanoylphorbol-13-acetate- and lysophosphatidic acid-induced signaling cascades. J Biol Chem 278: 17255–17262. 12611888 10.1074/jbc.M211835200

[8] Nanba D, Mammoto A, Hashimoto K, Higashiyama S (2003) Proteolytic release of the carboxy-terminal fragment of proHB-EGF causes nuclear export of PLZF. J Cell Biol 163: 489–502. 14597771 10.1083/jcb.200303017 PMC2173632

[9] Kinugasa Y, Hieda M, Hori M, Higashiyama S (2007) The carboxyl-terminal fragment of pro-HB-EGF reverses Bcl6-mediated gene repression. J Biol Chem 282: 14797–14806. 17392284 10.1074/jbc.M611036200

[10] Iwamoto R, Yamazaki S, Asakura M, Takashima S, Hasuwa H, et al (2003) Heparin-binding EGF-like growth factor and ErbB signaling is essential for heart function. Proc Natl Acad Sci U S A 100: 3221–3226. 12621152 10.1073/pnas.0537588100 PMC152273

[11] Jackson LF, Qiu TH, Sunnarborg SW, Chang A, Zhang C, et al (2003) Defective valvulogenesis in HB-EGF and TACE-null mice is associated with aberrant BMP signaling. EMBO J 22: 2704–2716. 12773386 10.1093/emboj/cdg264 PMC156761

[12] Mine N, Iwamoto R, Mekada E (2005) HB-EGF promotes epithelial cell migration in eyelid development. Development 132: 4317–4326. 16141218 10.1242/dev.02030

[13] Shirakata Y, Kimura R, Nanba D, Iwamoto R, Tokumaru S, et al (2005) Heparin-binding EGF-like growth factor accelerates keratinocyte migration and skin wound healing. J Cell Sci 118: 2363–2370. 15923649 10.1242/jcs.02346

[14] Das SK, Wang XN, Paria BC, Damm D, Abraham JA, et al (1994) Heparin-binding EGF-like growth factor gene is induced in the mouse uterus temporally by the blastocyst solely at the site of its apposition: a possible ligand for interaction with blastocyst EGF-receptor in implantation. Development 120: 1071–1083. 8026321 10.1242/dev.120.5.1071

[15] Raab G, Kover K, Paria BC, Dey SK, Ezzell RM, et al (1996) Mouse preimplantation blastocysts adhere to cells expressing the transmembrane form of heparin-binding EGF-like growth factor. Development 122: 637–645. 8625815 10.1242/dev.122.2.637

[16] Asakura M, Kitakaze M, Takashima S, Liao Y, Ishikura F, et al (2002) Cardiac hypertrophy is inhibited by antagonism of ADAM12 processing of HB-EGF: metalloproteinase inhibitors as a new therapy. Nat Med 8: 35–40. 11786904 10.1038/nm0102-35

[17] Powell PP, Klagsbrun M, Abraham JA, Jones RC (1993) Eosinophils expressing heparin-binding EGF-like growth factor mRNA localize around lung microvessels in pulmonary hypertension. Am J Pathol 143: 784–793. 8362977 PMC1887209

[18] Miyagawa J, Higashiyama S, Kawata S, Inui Y, Tamura S, et al (1995) Localization of heparin-binding EGF-like growth factor in the smooth muscle cells and macrophages of human atherosclerotic plaques. J Clin Invest 95: 404–411. 7814641 10.1172/JCI117669 PMC295446

[19] Peoples GE, Blotnick S, Takahashi K, Freeman MR, Klagsbrun M, et al (1995) T lymphocytes that infiltrate tumors and atherosclerotic plaques produce heparin-binding epidermal growth factor-like growth factor and basic fibroblast growth factor: a potential pathologic role. Proc Natl Acad Sci U S A 92: 6547–6551. 7604030 10.1073/pnas.92.14.6547 PMC41555

[20] Fu S, Bottoli I, Goller M, Vogt PK (1999) Heparin-binding epidermal growth factor-like growth factor, a v-Jun target gene, induces oncogenic transformation. Proc Natl Acad Sci U S A 96: 5716–5721. 10318950 10.1073/pnas.96.10.5716 PMC21926

[21] Kobrin MS, Funatomi H, Friess H, Buchler MW, Stathis P, et al (1994) Induction and expression of heparin-binding EGF-like growth factor in human pancreatic cancer. Biochem Biophys Res Commun 202: 1705–1709. 8060360 10.1006/bbrc.1994.2131

[22] Naef M, Yokoyama M, Friess H, Büchler MW, Korc M (1996) Co-expression of heparin-binding EGF-like growth factor and related peptides in human gastric carcinoma. Int J Cancer 66: 315–321. 8621250 10.1002/(SICI)1097-0215(19960503)66:3<315::AID-IJC8>3.0.CO;2-1

[23] Mishima K, Higashiyama S, Asai A, Yamaoka K, Nagashima Y, et al (1998) Heparin-binding epidermal growth factor-like growth factor stimulates mitogenic signaling and is highly expressed in human malignant gliomas. Acta Neuropathol 96: 322–328. 9796995 10.1007/s004010050901

[24] Thøgersen VB, Sørensen BS, Poulsen SS, Ørntoft TF, Wolf H, et al (2001) A subclass of HER1 ligands are prognostic markers for survival in bladder cancer patients. Cancer Res 61: 6227–6233. 11507076

[25] Miyamoto S, Hirata M, Yamazaki A, Kageyama T, Hasuwa H, et al (2004) Heparin-binding EGF-like growth factor is a promising target for ovarian cancer therapy. Cancer Res 64: 5720–5727. 15313912 10.1158/0008-5472.CAN-04-0811

[26] Tanaka Y, Miyamoto S, Suzuki SO, Oki E, Yagi H, et al (2005) Clinical significance of heparin-binding epidermal growth factor-like growth factor and a disintegrin and metalloprotease 17 expression in human ovarian cancer. Clin Cancer Res 11: 4783–4792. 16000575 10.1158/1078-0432.CCR-04-1426

[27] Hoffmann AC, Danenberg KD, Taubert H, Danenberg PV, Wuerl P (2009) A three-gene signature for outcome in soft tissue sarcoma. Clin Cancer Res 15: 5191–5198. 19671876 10.1158/1078-0432.CCR-08-2534

[28] Tsujioka H, Fukami T, Yotsumoto F, Ueda T, Hikita S, et al (2011) A possible clinical adaptation of CRM197 in combination with conventional chemotherapeutic agents for ovarian cancer. Anticancer Res 31: 2461–2465. 21873160

[29] Miyamoto S, Iwamoto R, Furuya A, Takahashi K, Sasaki Y, et al (2011) A novel anti-human HB-EGF monoclonal antibody with multiple antitumor mechanisms against ovarian cancer cells. Clin Cancer Res 17: 6733–6741. 21918176 10.1158/1078-0432.CCR-11-1029

[30] Keizer RJ, Huitema AD, Schellens JH, Beijnen JH (2010) Clinical pharmacokinetics of therapeutic monoclonal antibodies. Clin Pharmacokinet 49: 493–507. 20608753 10.2165/11531280-000000000-00000

[31] Buzzi S, Rubboli D, Buzzi G, Buzzi AM, Morisi C, et al (2004) CRM197 (nontoxic diphtheria toxin): effects on advanced cancer patients. Cancer Immunol Immunother 53: 1041–1048. 15168087 10.1007/s00262-004-0546-4 PMC11032786

[32] Khong TF, Fraser S, Katerelos M, Paizis K, Hill PA, et al (2000) Inhibition of heparin-binding epidermal growth factor-like growth factor increases albuminuria in puromycin aminonucleoside nephrosis. Kidney Int 58: 1098–1107. 10972674 10.1046/j.1523-1755.2000.00267.x

[33] Hamaoka M, Chinen I, Murata T, Takashima S, Iwamoto R, et al (2010) Anti-human HB-EGF monoclonal antibodies inhibiting ectodomain shedding of HB-EGF and diphtheria toxin binding. J Biochem 148: 55–69. 20332144 10.1093/jb/mvq033

[34] Tsuji I, Sato S, Otake K, Watanabe T, Kamada H, et al.. (2012) Characterization of a variety of neutralizing anti-heparin-binding epidermal growth factor-like growth factor monoclonal antibodies by different immunization methods. mAbs [Epub ahead of print]. 10.4161/mabs.21929 PMC3502240 23007682

[35] Thomas SM, Grandis JR, Wentzel AL, Gooding WE, Lui VW, et al (2005) Gastrin-releasing peptide receptor mediates activation of the epidermal growth factor receptor in lung cancer cells. Neoplasia 7: 426–431. 15967120 10.1593/neo.04454 PMC1501149

[36] Mitamura T, Higashiyama S, Taniguchi N, Klagsbrun M, Mekada E (1995) Diphtheria toxin binds to the epidermal growth factor (EGF)-like domain of human heparin-binding EGF-like growth factor/diphtheria toxin receptor and inhibits specifically its mitogenic activity. J Biol Chem 270: 1015–1019. 7836353 10.1074/jbc.270.3.1015

[37] Yu L, Liang XH, Ferrara N (2011) Comparing protein VEGF inhibitors: In vitro biological studies. Biochem Biophys Res Commun 408: 276–281. 21501594 10.1016/j.bbrc.2011.04.014

[38] Darling RJ, Brault PA (2004) Kinetic exclusion assay technology: characterization of molecular interactions. Assay Drug Dev Technol 2: 647–657. 15674023 10.1089/adt.2004.2.647

[39] Blake RCII, Delehanty JB, Khosraviani M, Yu H, Jones RM, et al (2003) Allosteric binding properties of a monoclonal antibody and its Fab fragment. Biochemistry 42: 497–508. 12525177 10.1021/bi0267339

[40] Abramovitch R, Neeman M, Reich R, Stein I, Keshet E, et al (1998) Intercellular communication between vascular smooth muscle and endothelial cells mediated by heparin-binding epidermal growth factor-like growth factor and vascular endothelial growth factor. FEBS Lett 425: 441–447. 9563510 10.1016/s0014-5793(98)00283-x

[41] Ushiro S, Ono M, Izumi H, Kohno K, Taniguchi N, et al (1996) Heparin-binding epidermal growth factor-like growth factor: p91 activation induction of plasminogen activator/inhibitor, and tubular morphogenesis in human microvascular endothelial cells. Jpn J Cancer Res 87: 68–77. 8609052 10.1111/j.1349-7006.1996.tb00202.x PMC5920984

[42] Hoskins JT, Zhou Z, Harding PA (2008) The significance of disulfide bonding in biological activity of HB-EGF, a mutagenesis approach. Biochem Biophys Res Commun 375: 506–511. 18725202 10.1016/j.bbrc.2008.08.062 PMC2632935

[43] Jin P, Zhang J, Beryt M, Turin L, Brdlik C, et al (2009) Rational optimization of a bispecific ligand trap targeting EGF receptor family ligands. Mol Med 15: 11–20. 19048033 10.2119/molmed.2008.00103 PMC2592073

[44] Brooke JS, Cha JH, Eidels L (1998) Diphtheria toxin:receptor interaction: association, dissociation, and effect of pH. Biochem Biophys Res Commun 248: 297–302. 9675130 10.1006/bbrc.1998.8953

[45] Goldstein NI, Prewett M, Zuklys K, Rockwell P, Mendelsohn J (1995) Biological efficacy of a chimeric antibody to the epidermal growth factor receptor in a human tumor xenograft model. Clin Cancer Res 1: 1311–1318. 9815926

[46] Hommel U, Harvey TS, Driscoll PC, Campbell ID (1992) Human epidermal growth factor. High resolution solution structure and comparison with human transforming growth factor alpha. J Mol Biol 227: 271–282. 1522591 10.1016/0022-2836(92)90697-i

[47] Ogiso H, Ishitani R, Nureki O, Fukai S, Yamanaka M, et al (2002) Crystal structure of the complex of human epidermal growth factor and receptor extracellular domains. Cell 110: 775–787. 12297050 10.1016/s0092-8674(02)00963-7

[48] Engler DA, Campion SR, Hauser MR, Cook JS, Niyogi SK (1992) Critical functional requirement for the guanidinium group of the arginine 41 side chain of human epidermal growth factor as revealed by mutagenic inactivation and chemical reactivation. J Biol Chem 267: 2274–2281. 1733935

[49] Koide H, Muto Y, Kasai H, Kohri K, Hoshi K, et al (1992) A site-directed mutagenesis study on the role of isoleucine-23 of human epidermal growth factor in the receptor binding. Biochim Biophys Acta 1120: 257–261. 1576151 10.1016/0167-4838(92)90245-9

[50] Tadaki DK, Niyogi SK (1993) The functional importance of hydrophobicity of the tyrosine at position 13 of human epidermal growth factor in receptor binding. J Biol Chem 268: 10114–10119. 8486681

[51] Jones JT, Ballinger MD, Pisacane PI, Lofgren JA, Fitzpatrick VD, et al (1998) Binding interaction of the heregulinbeta egf domain with ErbB3 and ErbB4 receptors assessed by alanine scanning mutagenesis. J Biol Chem 273: 11667–11674. 9565587 10.1074/jbc.273.19.11667

[52] Cook PW, Mattox PA, Keeble WW, Pittelkow MR, Plowman GD, et al (1991) A heparin sulfate-regulated human keratinocyte autocrine factor is similar or identical to amphiregulin. Mol Cell Biol 11: 2547–2557. 2017164 10.1128/mcb.11.5.2547-2557.1991 PMC360024

[53] Inui S, Higashiyama S, Hashimoto K, Higashiyama M, Yoshikawa K, et al (1997) Possible role of coexpression of CD9 with membrane-anchored heparin-binding EGF-like growth factor and amphiregulin in cultured human keratinocyte growth. J Cell Physiol 171: 291–298. 9180898 10.1002/(SICI)1097-4652(199706)171:3<291::AID-JCP7>3.0.CO;2-J

[54] Nakagawa M, Nabeshima K, Asano S, Hamasaki M, Uesugi N, et al (2009) Up-regulated expression of ADAM17 in gastrointestinal stromal tumors: coexpression with EGFR and EGFR ligands. Cancer Sci 100: 654–662. 19298600 10.1111/j.1349-7006.2009.01089.x PMC11158838

[55] McIntyre E, Blackburn E, Brown PJ, Johnson CG, Gullick WJ (2010) The complete family of epidermal growth factor receptors and their ligands are co-ordinately expressed in breast cancer. Breast Cancer Res Treat 122: 105–110. 19760033 10.1007/s10549-009-0536-5

[56] Ruck A, Paulie S (1998) EGF, TGF alpha, AR and HB-EGF are autocrine growth factors for human bladder carcinoma cell lines. Anticancer Res 18: 1447–1452. 9673354

[57] O-Charoenrat P, Rhys-Evans P, Eccles S (2000) Expression and regulation of c-ERBB ligands in human head and neck squamous carcinoma cells. Int J Cancer 88: 759–765. 11072245 10.1002/1097-0215(20001201)88:5<759::aid-ijc12>3.0.co;2-0

[58] Tørring N, Jørgensen PE, Sørensen BS, Nexø E (2000) Increased expression of heparin binding EGF (HB-EGF), amphiregulin, TGF alpha and epiregulin in androgen-independent prostate cancer cell lines. Anticancer Res 20: 91–95. 10769639

[59] Yotsumoto F, Yagi H, Suzuki SO, Oki E, Tsujioka H, et al (2008) Validation of HB-EGF and amphiregulin as targets for human cancer therapy. Biochem Biophys Res Commun 365: 555–561. 18023415 10.1016/j.bbrc.2007.11.015

